# Gastrointestinal Stromal Tumors: Deception to the Eyesight

**DOI:** 10.7759/cureus.1531

**Published:** 2017-07-31

**Authors:** Fady G. Haddad, Magda Daoud, Mayurathan Kesavan, Sherif Andrawes

**Affiliations:** 1 Department of Internal Medicine, Staten Island University Hospital; 2 Department of Gastroenterology, Staten Island University Hospital

**Keywords:** gastrointestinal stromal tumors

## Abstract

Gastrointestinal stromal tumors (GISTs) are rare soft tissue tumors. Despite their rarity, these tumors are the most common gastrointestinal (GI) mesenchymal tumors. They can involve various parts of the gastrointestinal tract. GISTs growth can be intramural, intraluminal or exophytic. Symptoms are usually related to GI bleeding and to adjacent organ compression by the tumor. Endoscopy can suggest the diagnosis, but tissue sampling is required for the diagnosis. Herein, we present a unique case of GIST where the patient had negative endoscopic findings despite the large size of the tumor, thus abdominal computed tomography scan and endoscopic ultrasound was required to make the diagnosis.

## Introduction

Gastrointestinal stromal tumors (GISTs) are rare tumors with malignant potential that can involve various parts of the gastrointestinal (GI) tract [1-2]. They mostly present with GI bleeding and symptoms related to adjacent organ compression by the tumor, however, patients can be asymptomatic. The GISTs growth pattern can be intramural, intraluminal and exophytic [3]. We present a unique case of GIST where despite the large size of the tumor, it lacked suggestive endoscopic manifestations. Thus, there was the need for abdominal computed tomography (CT) scan and endoscopic ultrasound (EUS) to establish the diagnosis. Informed consent was obtained from the patient for this study

## Case presentation

**Figure 1:**
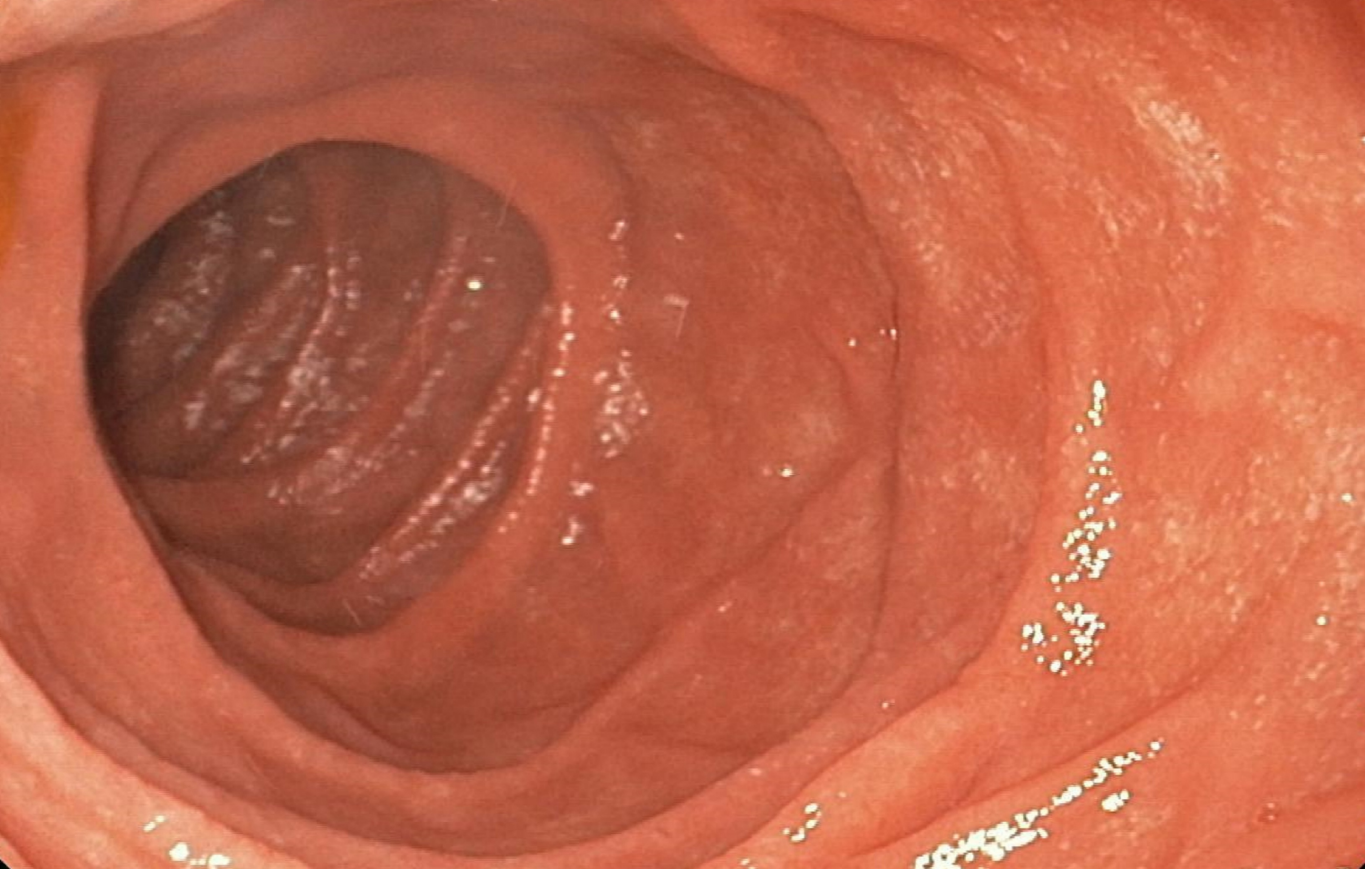
Upper endoscopy revealing the absence of evident mucosal or submucosal lesion in the duodenal lumen.

A 51-year-old healthy male presented with intermittent epigastric pain of few weeks duration. He denied having weight loss or any additional symptoms. His physical examination and blood tests were within normal range. Esophagogastroduodenoscopy (EGD) revealed no luminal abnormalities (Figure 1). 

**Figure 2:**
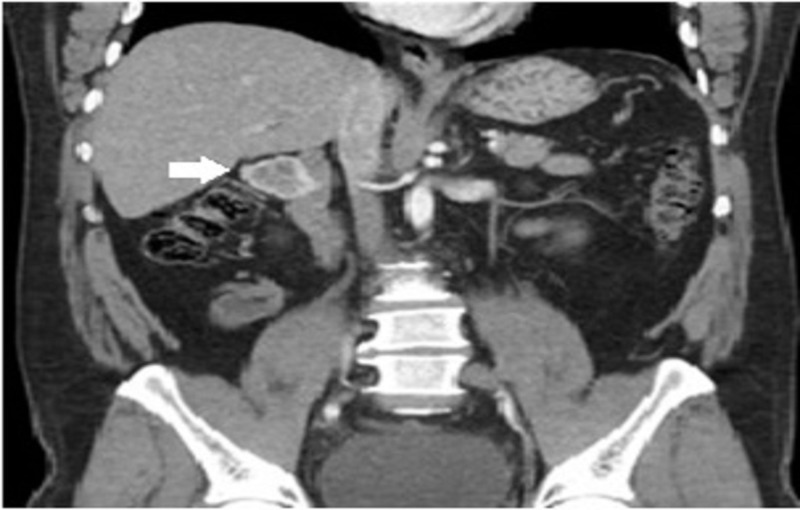
Coronal view of contrast computed tomography scan of the abdomen showing a 3.5 cm mass (white arrow) located laterally to the second part of the duodenum, anteriorly to the right kidney and posteriorly to the hepatic flexure. The mass was separate from the adrenal gland and demonstrated a heterogeneous ring enhancement and contained some coarse calcifications anteriorly.

A contrast abdominal computed tomography (CT) scan showed a 3.5 cm mass lateral to the duodenum, demonstrating a heterogeneous ring enhancement and containing some coarse calcifications anteriorly (Figure 2). 

**Figure 3:**
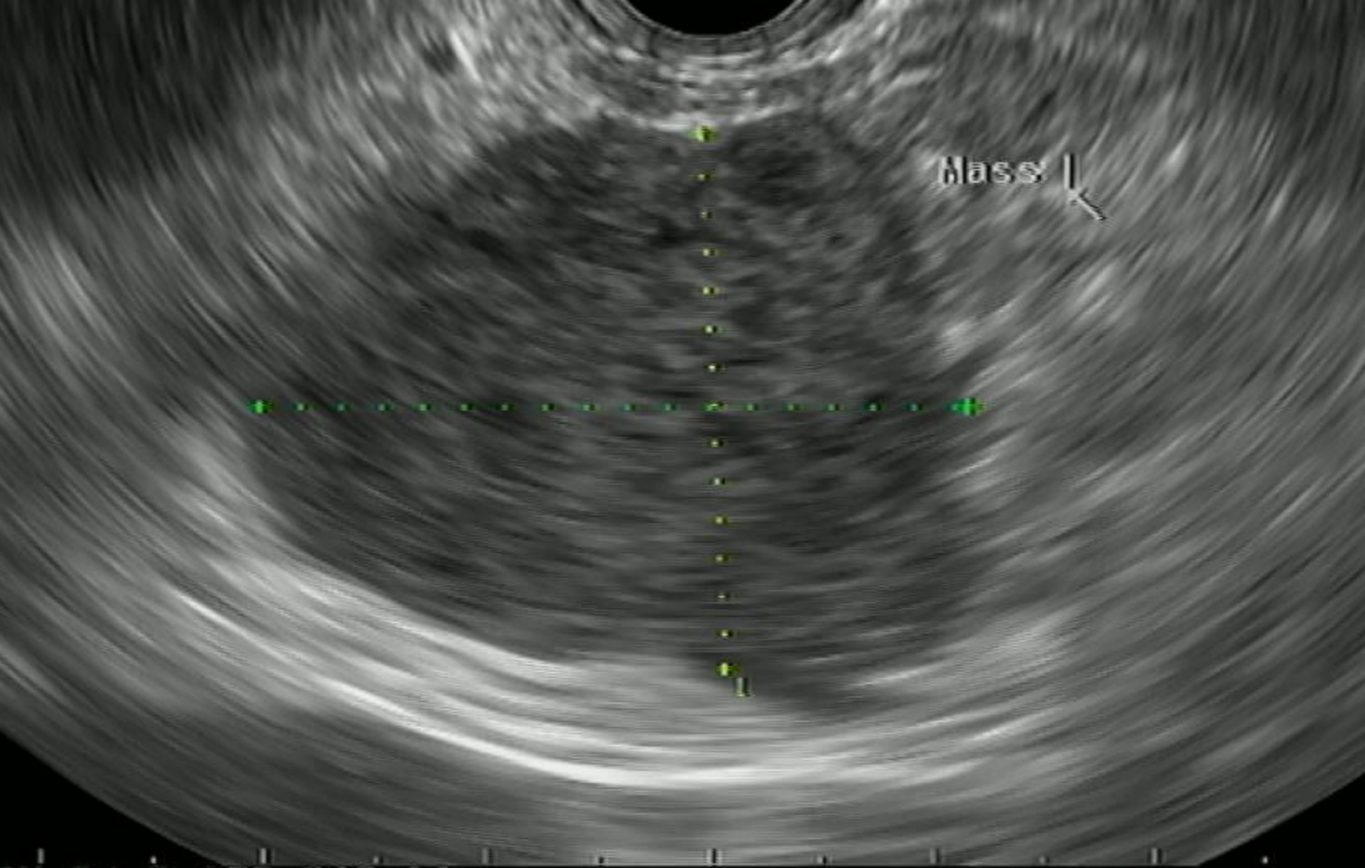
Endoscopic ultrasound showing a 6 cm x 3.5 cm circumferential lesion located outside the lumen between the first and second segments of the duodenum. The lesion was hypoechoic, homogenous with central necrosis and microcystic spaces and originated from the muscularis propria of the duodenum.

EUS showed a 6 cm x 3.5 cm circumferential lesion between the first and second segments of the duodenum (Figure 3).

**Figure 4:**
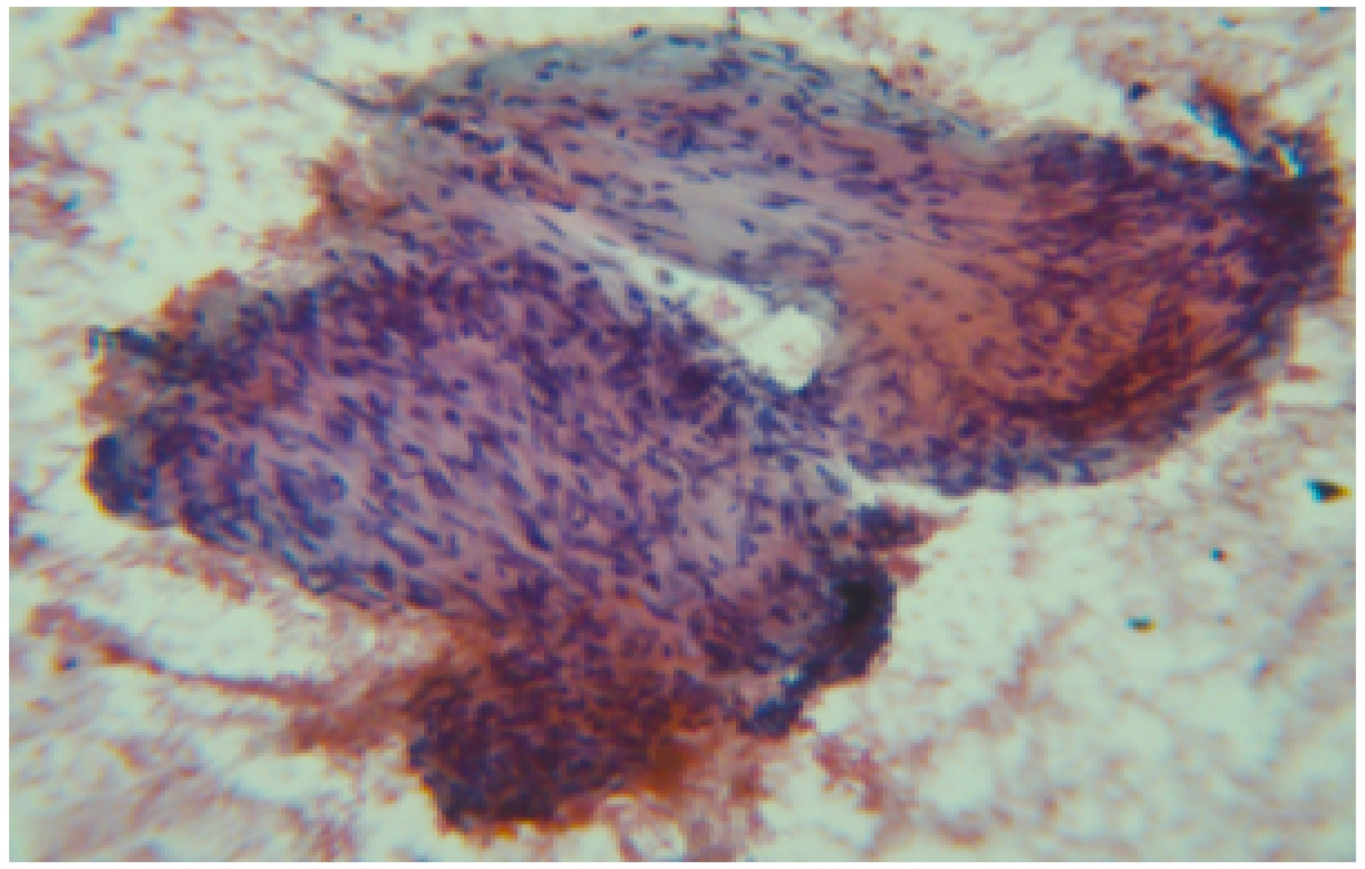
Papanicolaou stain-20 x cytology of the fine needle aspiration specimen showing few clusters of atypical spindle cells.

The lesion was hypoechoic, homogeneous with central necrosis and micro cystic spaces, and originated from the muscularis propria of the duodenum. Fine needle aspiration (FNA) revealed few clusters of atypical spindle cells (Figure 4). 

**Figure 5:**
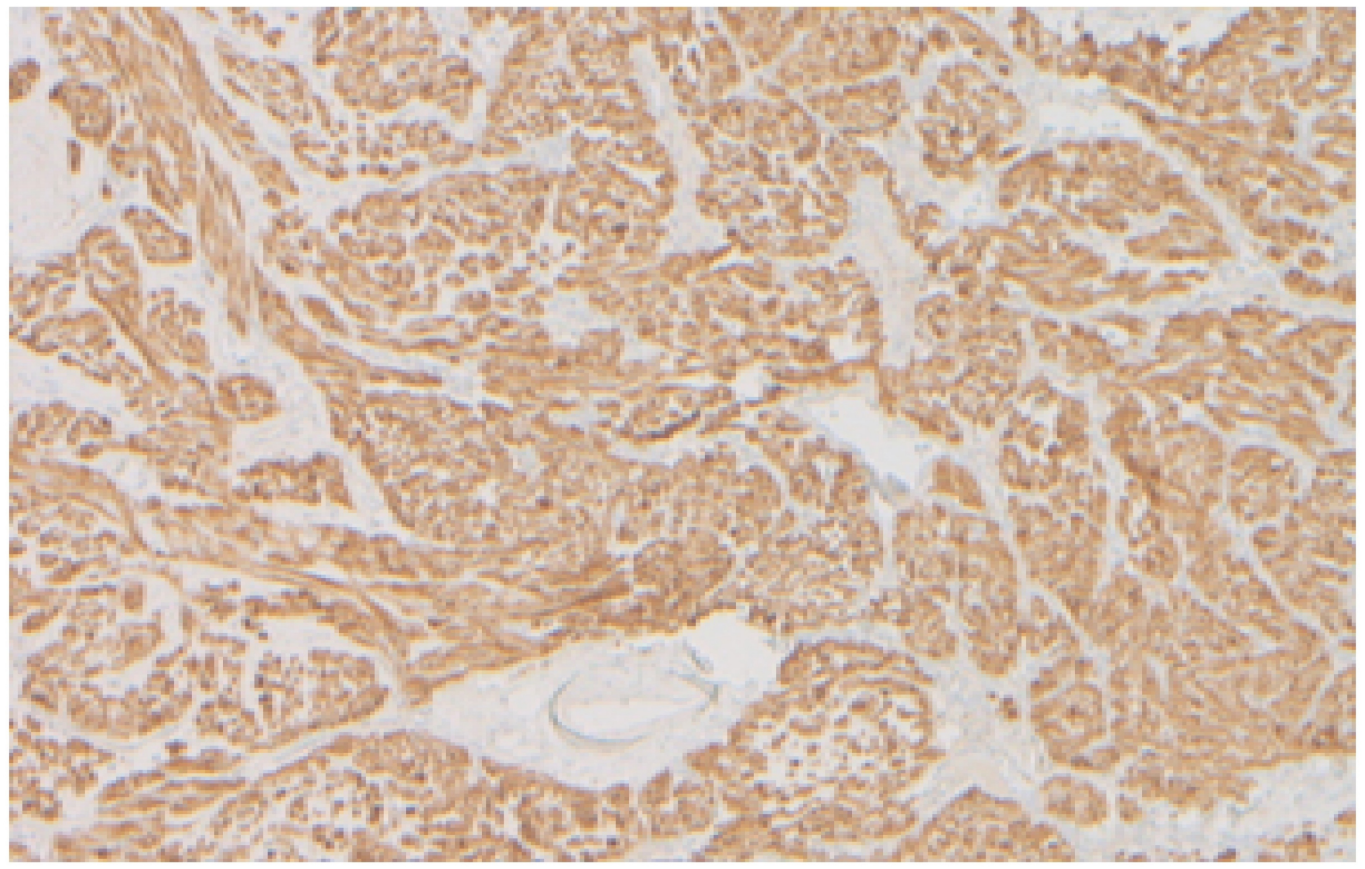
Hematoxylin and eosin stain-20 x of the surgical specimen showing spindle shaped cells with a mitotic rate

The patient underwent surgical resection of the mass. The tumor had a low histologic grade (mitotic rate <5/50 high power field and a primary tumor pathologic stage pT2 (Figure 5). 

**Figure 6:**
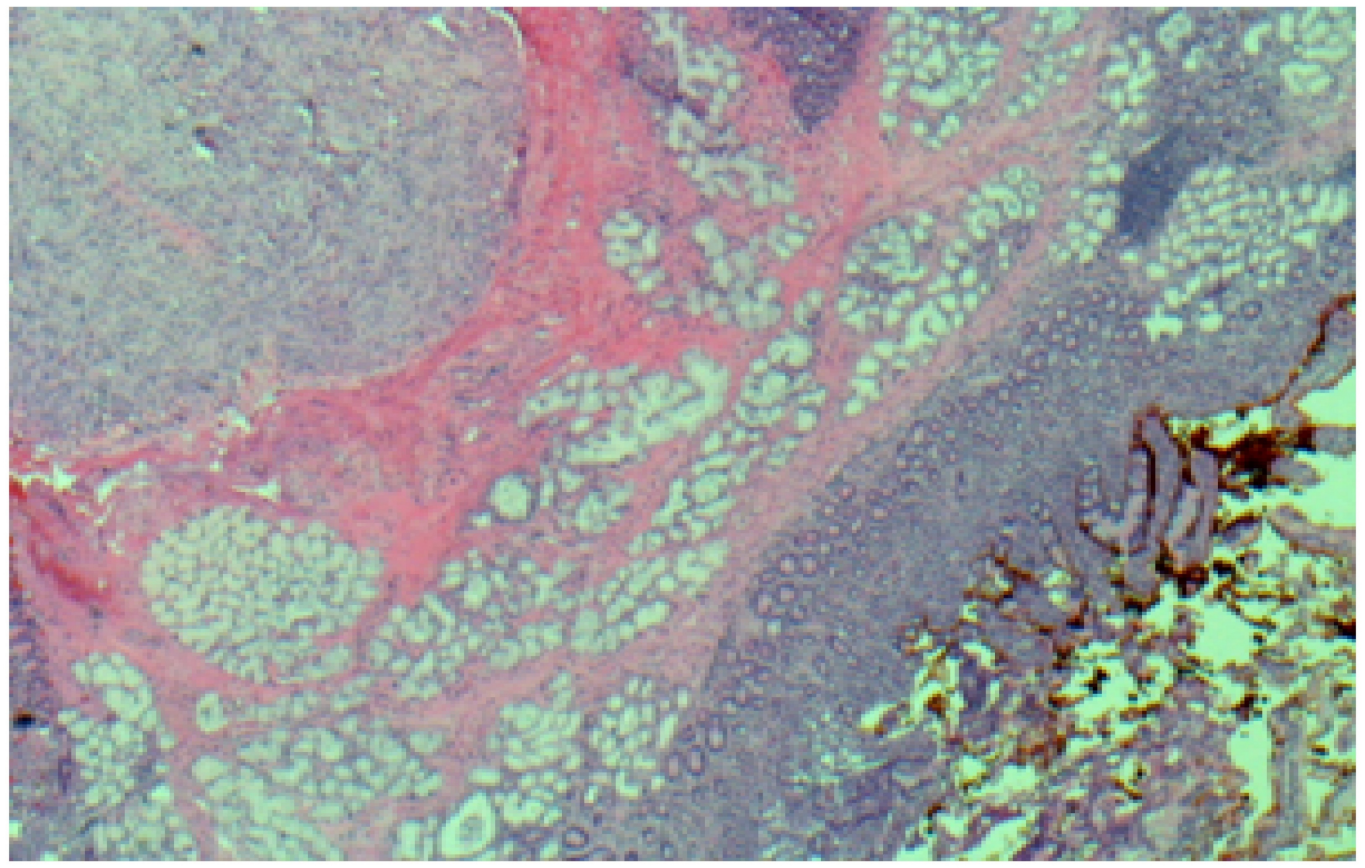
Immunohistochemistry- 4 x cellular staining showing strong positivity for CD117/c-Kit.

The proliferative index Ki67 was < 5%. Cellular staining was strongly positive for CD117/c-Kit (Mast/stem cell growth factor receptor -SCFR) confirming the diagnosis of spindle cell type gastrointestinal (GI) stromal tumor (Figure 6).

**Figure 7:**
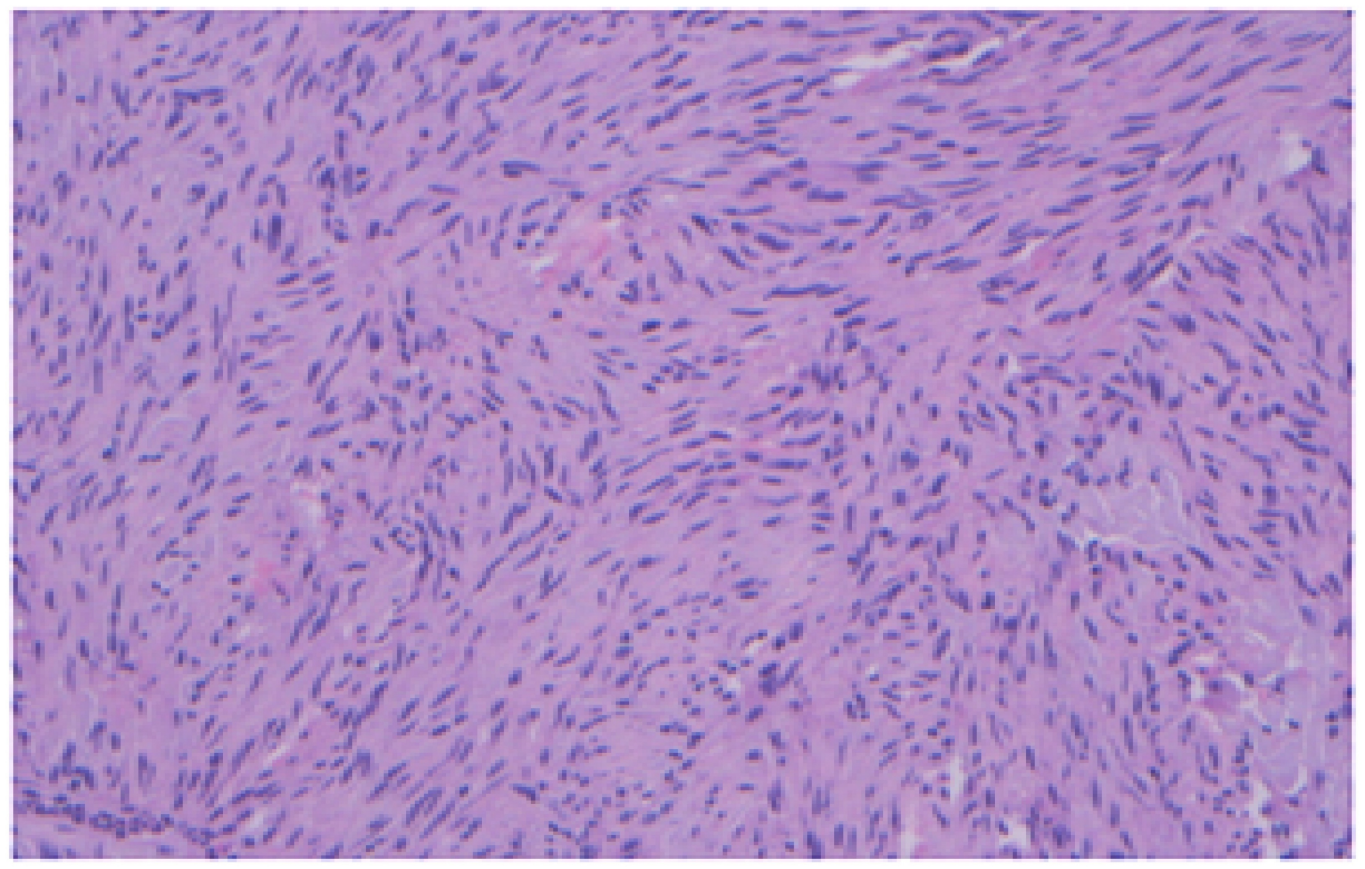
Hematoxylin and eosin stain- 4 x showing a partial invasion of the muscularis propria by the tumor and focal erasing of the anteriorly overlying duodenal mucosa with ulcer formation.

The cells also stained weakly for smooth muscle actin (SMA), but not for smooth muscle myosin (SMM), desmin or S100. Further findings included partial invasion of the adjacent muscularis propria and focal erasing of the anteriorly overlying duodenal mucosa with ulcer formation, however, the resection margins were free of tumor (Figure 7).

## Discussion

The GISTs are uncommon GI tumors that involve mostly the stomach (60-70%) followed by the small intestine (20-30%) [1-2]. Despite their rarity, these tumors are the most common GI mesenchymal tumors [1]. The GISTs growth pattern can be intramural, intraluminal and exophytic [3]. Exophytic lesions are the most common and occur in 68-79% cases [4]. Symptoms occur in the majority of cases and are usually related to gastrointestinal bleeding and to adjacent organ compression by the tumor [1, 5]. However, asymptomatic GISTs account for 10-30% of total cases, which is explained by their submucosal localization and nonaggressive behavior [1-2]. Computed tomography scan and endoscopy suggests the diagnosis, whereas endoscopic ultrasound and/or surgery offer a pathological confirmation through demonstration of positive KIT (CD117) or platelet- derived growth factor receptor alpha (PDGFRA) gene mutations [1, 6]. Treatment for symptomatic GISTs relies on surgical resection, whereas the management of asymptomatic GISTs is still controversial [1-2]. Additional studies are required to further characterize these tumors.

## Conclusions

We present a unique case of a large gastrointestinal stromal tumor with negative endoscopic findings, thus was the need for abdominal computed tomography scan and endoscopic ultrasound to suggest the diagnosis. An increased awareness of gastrointestinal stromal tumors and their malignant potential is required to prevent devastating complications.
